# DISPARITIES AMONG LONG-TERM AND SHORT-TERM USERS OF MEALS ON WHEELS SERVICES

**DOI:** 10.1093/geroni/igae098.4276

**Published:** 2024-12-31

**Authors:** France Weaver, Jennifer Chubinski, Sarah Walsh

**Affiliations:** West Virginia University, Morgantown, West Virginia, United States; Xavier University, Cincinnati, Ohio, United States; Eastern Michigan University, Ypsilanti, Michigan, United States

## Abstract

Home-delivered meal programs for older adults, commonly known as Meals on Wheels (MOW) services, provide nutritional benefits and are valued by clients, but most studies capture use over a short period of time, typically a few months. This study is the first to focus on duration of use and identify disparities in the long-term and short-term MOW users. It uses 35,756 observations from the 2013-2022 National Health and Aging Trends Study (NHATS), an annual survey of Medicare enrollees aged 65+. A fixed effect multinomial logit model, with complex survey design, predicts the probabilities of being a short-term or long-term MOW user across lagged sociodemographic and health characteristics. Short-term users are defined as reporting MOW use once (12.4% of the sample) and the long-term users indicate use for 2+ years (8.6%). Persons who are Black are statistically more likely to be long-term MOW users (13.7%) than non-Black (8.0%), similarly for those on Medicaid (15.7%), having 2+ instrumental activity of daily living limitations (10.9%), and pre-frail (9.5%). In contrast, no racial or health differences are found for short-term MOW users, except for those on Medicaid (18.7%). These results identify the populations more likely to be long-term MOW users, but further research is needed to determine if prolonged MOW use is an indicator of sustained independence or a consequence of barriers to additional services. This study helps to better understand the role played by MOW and the federal Older Americans Act Nutrition Program, which funds those services, at serving diverse populations.

